# High quality draft genome sequence of *Staphylococcus cohnii subsp. cohnii* strain hu-01

**DOI:** 10.4056/sigs.5429581

**Published:** 2014-03-15

**Authors:** XinJun Hu, Ang Li, LongXian Lv, Chunhui Yuan, Lihua Guo, Xiawei Jiang, Haiyin Jiang, GuiRong Qian, BeiWen Zheng, Jing Guo, LanJuan Li

**Affiliations:** a State Key Laboratory for Diagnosis and Treatment of Infectious Disease, The First Affiliated Hospital, Zhejiang University, Hangzhou, PR China.; b Collaborative Innovation Center for Diagnosis and Treatment of Infectious Diseases, Hangzhou, China

**Keywords:** *Staphylococcus cohnii subsp. cohnii*, genome, Hiseq2000

## Abstract

*Staphylococcus cohnii subsp. cohnii* belongs to the family *Staphylococcaceae* in the order *Bacillales*, class *Bacilli* and phylum *Firmicutes*. The increasing relevance of *S. cohnii* to human health prompted us to determine the genomic sequence of *Staphylococcus cohnii subsp. cohnii* strain hu-01, a multidrug-resistant isolate from a hospital in China. Here we describe the features of *S. cohnii subsp. cohnii* strain hu-01, together with the genome sequence and its annotation. This is the first genome sequence of the species *Staphylococcus cohnii*.

## Introduction

*Staphylococcus cohnii* belongs to the Coagulase-Negative Staphylococci group. It was described by Schleifer and Kloos (1975) and was named for Ferdinand Cohn, a German botanist and bacteriologist [1]. Recently, more cases of *Staphylococcus cohnii* infection have been reported in the literature. This organism may be responsible for brain abscess, pneumonia, acute cholecystitis, endocarditis, bacteremia, urinary tract infection and septic arthritis [2]. *S. cohnii* is comprised of two subspecies that are defined on the basis of their phenotypic characteristics: *Staphylococcus cohnii subsp. cohnii* and *Staphylococcus cohnii subsp. urealyticus* [3]. *S. cohnii subsp. cohnii is a* Gram-positive coccus, coagulase negative and catalase positive, that behaves like a commensal mucocutaneous bacterium [4]. It has more frequently been isolated in hospital than in non-hospital environments [2]. Here we report this draft genome of *S. cohnii subsp. cohnii* strain hu-01, the first genome of this species to be sequenced.

## Classification and features

**Figure1:**
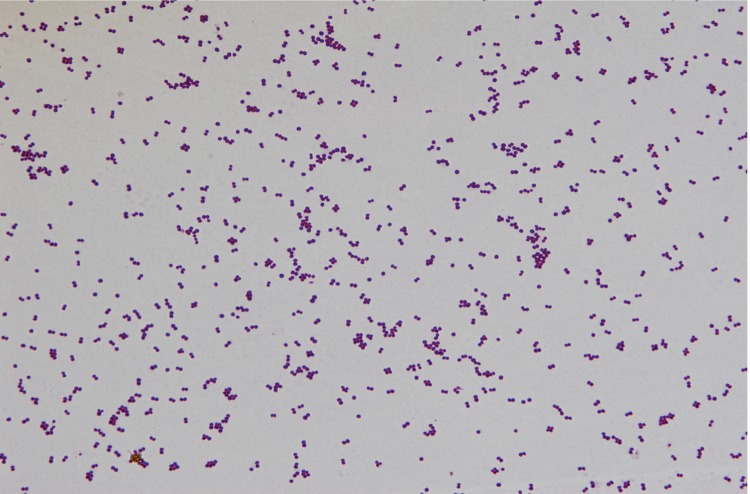
Gram staining of *S. cohnii subsp. cohnii* strain hu-01

**Figure 2:**
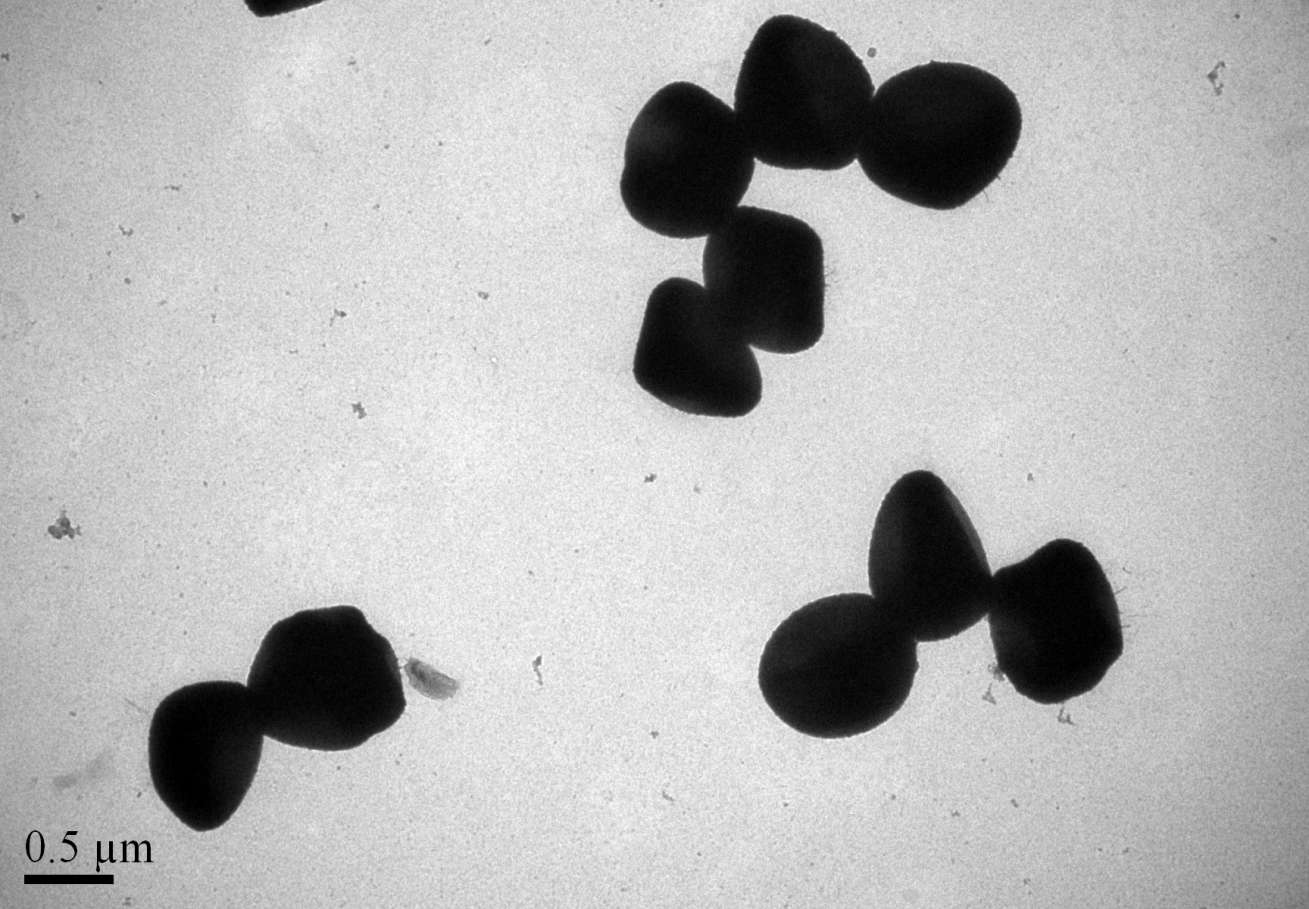
Transmission electron micrograph of cells of strain hu-01. Bar: 0.5 µm

Strain hu-01 was isolated from a hospital environment in Zhejiang province, China, in October 2012. It is a Gram-positive, coccus-shaped bacterium that can grow on 5% sheep blood enriched Columbia agar (BioMérieux, Marcyl’Etoile, France) at 37°C. Growth occurs under either aerobic or anaerobic conditions. The optimum temperature for growth is 37 ºC, with a temperature range of 15-45 ºC (Table 1). Cell morphology, motility and sporulation were examined by using transmission electron (H-600, Hitachi) microscopy. Cells of strain hu-01 are coccoidal, 0.6 to 1.2 μm in diameter, occurring predominantly singly or in pairs (Figure 1 and Figure 2).

**Table1:** Classification and general features of *S. cohnii subsp. cohnii* strain hu-01 according to the MIGS recommendations [9].

**MIGS ID**	**Property**	**Term**	**Evidence code^a^**
		Domain *Bacteria*	TAS [20]
		Phylum *Firmicutes*	TAS [21-23]
		Class *Bacilli*	TAS [24,25]
	Current classification	Order *Bacillales*	TAS [26,27]
		Family *Staphylococcaceae*	TAS [24,28]
		Genus *Staphylococcus*	TAS [26,29-31]
		Species *Staphylococcus cohnii subsp. cohnii*	TAS [1,3]
		Strain hu-01	IDA
	Gram stain	Positive	IDA
	Cell shape	coccus	IDA
	Motility	Nonmotile	IDA
	Sporulation	Nonsporulating	IDA
	Temperature range	15-45°C	IDA
	Optimum temperature	37°C	IDA
MIGS-6.3	Salinity	Tolerates 10% NaCl	IDA
MIGS-22	Oxygen	Facultatively anaerobic	IDA
	Carbon source	D-mannitol, fructose, trehalose	IDA
	Energy source	fructose, trehalose	IDA
MIGS-6	Habitat	Hospital environment	IDA
MIGS-15	Biotic relationship	Free living	IDA
MIGS-14	Pathogenicity	Opportunistic pathogen	IDA
	Isolation	Hospital	IDA
MIGS-4	Geographic location	Hangzhou, China	IDA
MIGS-5	Sample collection time	October, 2012	IDA
MIGS-4.1	Latitude	30°16’N	IDA
MIGS-4.2	Longitude	120°12’E	IDA
MIGS-4.3	Depth	unknown	IDA
MIGS-4.4	Altitude	50 (meters)	IDA

^a^Evidence codes-IDA: Inferred from Direct Assay; TAS: Traceable Author Statement (i.e., a direct report exists in the literature); NAS: Non-traceable Author Statement (i.e., not directly observed for the living, isolated sample, but based on a generally accepted property for the species, or anecdotal evidence). These evidence codes are from the Gene Ontology project [32]. If the evidence code is IDA, then the property should have been directly observed, for the purpose of this specific publication, for a live isolate by one of the authors, or an expert or reputable institution mentioned in the acknowledgements.

**Figure 3:**
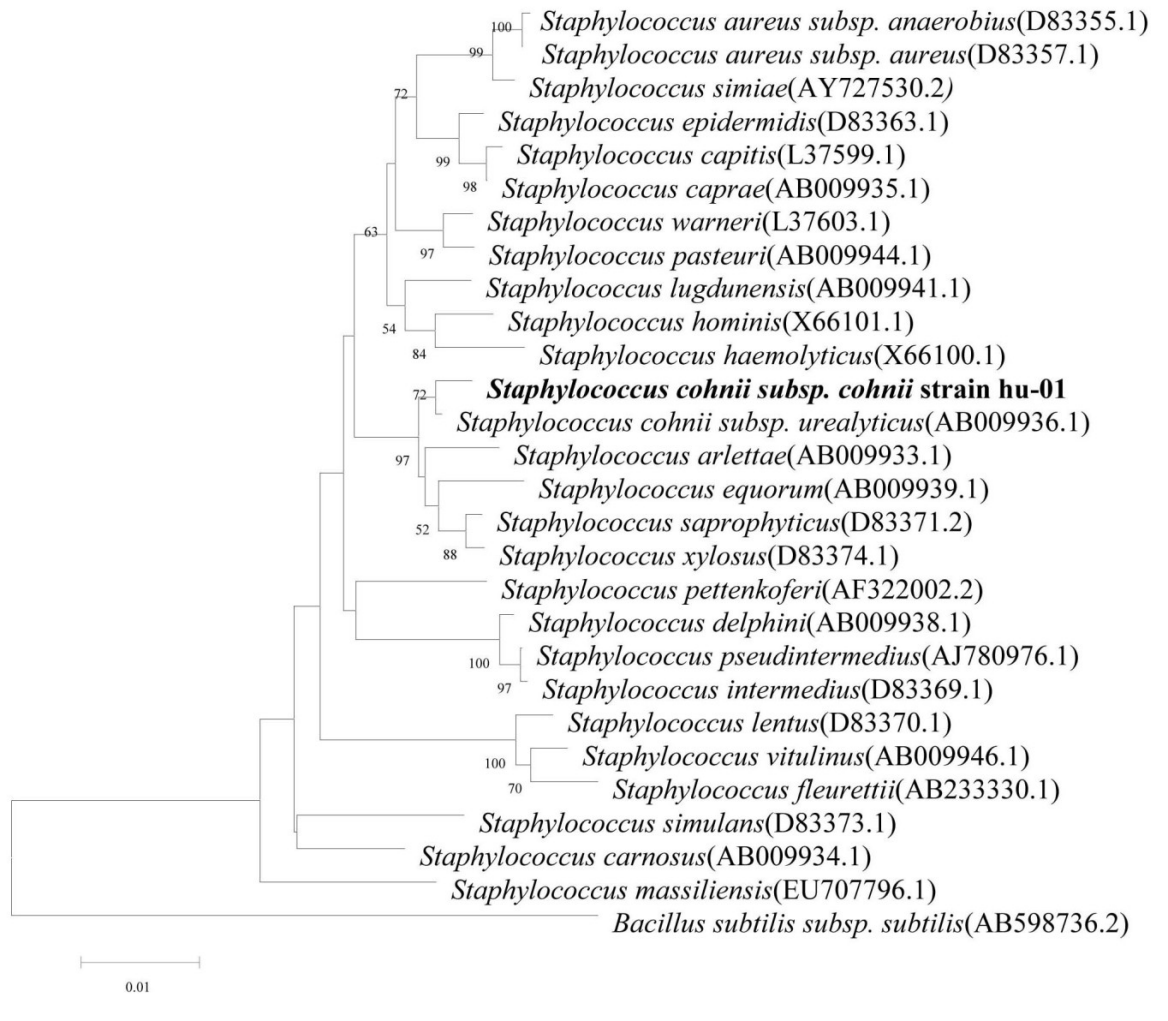
Phylogenetic tree depicting the relationship between *S. cohnii subsp. cohnii strain hu-01* and other members of the genus *Staphylococcus*. The strains and their corresponding Genbank accession numbers are shown following the organism name and indicated in parentheses. The phylogenetic tree uses 16S rRNA gene sequences aligned by the CLUSTALW [7], and phylogenetic inferences were made using Neighbor-joining method based on Kimura 2-parameter model within the MEGA5 software [8] and rooted with *Bacillus subtilis subsp. subtilis*. Bootstrap consensus trees were inferred from 100 replicates, only bootstrap values > 50% were indicated.

Comparative 16S rRNA gene sequence analysis by BLASTN [5,6] using the NCBI-NR/NT database revealed 94-99% sequence similarity to members of genus *Staphylococcus*. Neighbor-Joining phylogenetic analysis based on Kimura 2-parameter model indicated the strain hu-01 is most closely related the strain *Staphylococcus cohnii subsp. urealyticus* (AB009936.1) (Figure 3).

Biochemical features were tested by using two automated systems, the Vitek2 Compact (bioMérieux, Marcy l'Etoile, France) and Phoenix 100 ID/AST system (Becton Dickinson Company [BD], Sparks, Maryland, USA). Positive reactions were obtained for D-fructose, trehalose, D-gluconic acid and D-mannitol. Negative reactions were observed for glucose, D-trehalose, D-sucrose, maltose, urea, cellobiose, glucoside, D-tagatose and maltotriose. This strain was susceptible to gentamicin, ciprofloxacin, levofloxacin, moxifloxacin, quinupristin, linezolid, vancomycin, tetracycline, tigecycline, nitrofurantoin, rifampicin, trimethoprim and resistant to cefoxitin, benzylpenicillin, oxacillin, erythromycin, clindamycin.

## Genome sequencing information

### Genome project history

*S. conhii subsp. cohnii* strain hu-01 was selected for sequencing because of its increasing relevance to human health. The strain was isolated from a hospital environment in China. This whole genome shotgun project of *S. conhii subsp. cohnii* strain hu-01 was deposited at DDBJ/EMBL/GenBank under the accession AYOS00000000. Table 2 presents the project information and its association with MIGS version 2.0 compliance [9].

**Table 2:** Project information

**MIGS ID**	**Property**	**Term**
MIGS-31	Finishing quality	High-quality draft
MIGS-28	Libraries used	One pair-end 500 bp library
MIGS-29	Sequencing platforms	Illumina HiSeq 2000
MIGS-31.2	Fold coverage	150×(based on 500 bp library)
MIGS-30	Assemblers	Velvet 1.2.07
MIGS-32	Gene calling method	Glimmer 3.0
	Genbank ID	AYOS00000000
	Genbank Date of Release	Jan 06, 2014
	GOLD ID	Gi0062613
MIGS-13	Project relevance	Biotechnology, Pathway, Pathogenic

### Growth conditions and DNA isolation

*S. conhii subsp .cohnii* strain hu-01 was grown aerobically on Columbia blood agar base, at 37°C for 24h. Genomic DNA was extracted using the DNeasy blood and tissue kit (Qiagen, Germany), according to the manufacturer’s recommended protocol. The quantity of DNA was measured by the NanoDrop Spectrophotometer and Cubit. Then 10μg of DNA was sent to the State Key Laboratory for Diagnosis and Treatment of Infectious Disease at Zhejiang University for sequencing on a Hiseq2000 (Illumina, CA) sequencer.

### Genome sequencing and assembly

One DNA library was generated (500 bp insert size, with the Illumina adapter at both ends, detected by Agilent DNA analyzer 2100), then sequencing was performed by using an Illumina Hieseq 2000 genomic sequencer, with a 2×100 pair end sequencing strategy. A total of 1,103 M bp of sequence data was produced which was assessed for quality by the following criteria: 1) Reads linked to adapters at both end were considered as sequencing artifacts then removed. 2) Bases with a quality index lower than Q20 at both ends were trimmed. 3) Reads with ambiguous bases (N) were removed. 4) Single qualified reads were discarded (In this situation, one read is qualified but its mate is not). A total of 867.94 M clean filtered reads were assembled into scaffolds using the Velvet version 1.2.07 with parameters "-scaffolds no" [10], then we used a PAGIT flow [11] to prolong the initial contigs and correct sequencing errors. to arrive at a set of improved scaffolds.

### Genome annotation

Predict genes were identified using Glimmer version 3.0 [12],tRNAscan-SE version 1.21 [13] was used to find tRNA genes, whereas ribosomal RNAs were found by using RNAmmer version 1.2 [14]. To annotate predicted genes, we used HMMER version 3.0 [15], with parameters 'hmmscan -E 0.01 -domainE 0.01' to align genes against Pfam version 27.0 [16] (only pfam-A was used) to find genes with conserved domains. The KAAS server [17] was used to assign translated amino acids (with genetic code table 11) into KEGG Orthology with SBH (single-directional best hit) method. Translated genes were aligned with the COG database using NCBI blastp (hits should have scores no less than 60, e-value is no more than 1e-6). To find genes with hypothetical or putative function, we aligned genes against NCBI nucleotide sequence database (nt database was downloaded at Sep 20, 2013) by using NCBI blastn, only if hits have an identity of no less than 0.95, coverage no less than 0.9, and the reference gene had an annotation of putative or hypothetical. To define genes with signal peptide, we use signalp version 4.1 [18] to identify genes with signal peptide with default parameters except " -t gram+ ". TMHMM2.0 [19] was used to identify genes with transmembrane helices.

## Genome properties

The draft genome sequence of *S. conhii subsp. cohnii* strain hu-01 revealed a genome size of 5,761,489 bp and a G+C content of 34.85% (521 scaffolds with N50 is 39,926 bp). These scaffolds contain 5,820 coding sequences (CDSs), 61 tRNAs (excluding 6 Pseudo tRNAs) and incomplete rRNA operons (10 small subunit rRNA and 3 large subunit rRNAs). A total of 1,840 protein-coding genes were assigned as putative function or hypothetical proteins. 3,734 genes were categorized into COGs functional groups. The properties and the statistics of the genome are summarized in Table 3 and Table 4.

**Table 3:** Genome statistics of *S. cohnii subsp. cohnii* strain hu-01

**Attribute**	**Value**	**% of total^a^**
Genome size (bp)	5,761,489	--
DNA coding region (bp)	4,751,472	82.469
DNA G+C content (bp)	1,697,984	29.471
Total genes	5,833	--
RNA genes	13	0.221
Protein-coding genes	5,820	99.777
Genes with function prediction	1,840	31.544
Genes assigned to COGs	3,734	64.015
Genes assigned to Pfam domains	4,943	84.741
Genes with signal peptides	431	7.388
Genes with transmembrane helices	1,629	27.927

a) The total is based on either the size of the genome in base pairs or the total number of genes in the annotated genome.

**Table 4:** Number of genes associated with the general COG functional categories

**Code**	**Value^a^**	**%age^b^**	**Description**
J	230	3.95	Translation, ribosomal structure and biogenesis
K	452	7.77	Transcription
L	184	3.16	Replication, recombination and repair
B	3	0.05	Chromatin structure and dynamics
D	72	1.24	Cell cycle control, cell division, chromosome partitioning
V	187	3.21	Defense mechanisms
T	238	4.09	Signal transduction mechanisms
M	254	4.36	Cell wall/membrane/envelope biogenesis
N	70	1.20	Cell motility
Z	1	0.02	Cytoskeleton
W	1	0.02	Extracellular structures
U	57	0.98	Intracellular trafficking, secretion, and vesicular transport
O	147	2.53	Posttranslational modification, protein turnover, chaperones
C	292	5.02	Energy production and conversion
G	384	6.60	Carbohydrate transport and metabolism
E	640	11.0	Amino acid transport and metabolism
F	140	2.41	Nucleotide transport and metabolism
H	234	4.02	Coenzyme transport and metabolism
I	165	2.84	Lipid transport and metabolism
P	389	6.68	Inorganic ion transport and metabolism
Q	197	3.38	Secondary metabolites biosynthesis, transport and catabolism
R	841	14.45	General function prediction only
S	403	6.92	Function unknown
--^c^	483	8.30	Not archived in COGs
--^d^	1603	27.54	No hits

a) For some genes, qualified alignments can occur with several genes belonging to different COG categories. In such cases only the best match to a single COG category is considered. b) The total is based on the total number of protein coding genes(5,820) in the annotated genome. c) These genes have alignments with reference genes archived in COG, but these reference genes do not have COG categories. d) Genes without a qualified hit to a reference genes.

## Conclusion

*Staphylococcus cohnii ssp. cohnii* are part of the normal flora of human skin and mucous membranes which, in particular conditions, may become opportunistic pathogens [4]. The genome sequence of *Staphylococcus cohnii subsp. cohnii* strain hu-01 will provide the basis to elucidate the molecular principles of host colonization and insight into the genetic background of this organism’s pathogenesis.
